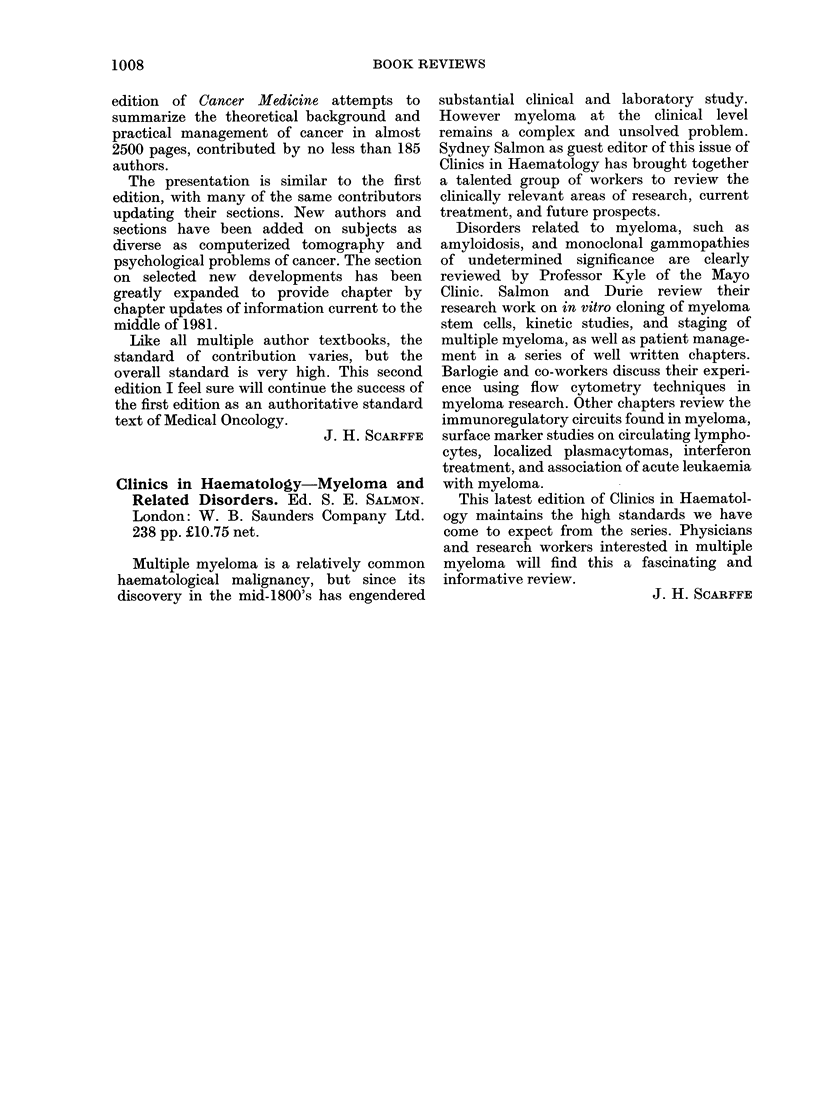# Clinics in Haematology—Myeloma and Related Disorders

**Published:** 1982-12

**Authors:** J. H. Scarffe


J. H. SCARFFE

**Clinics in Haematology—Myeloma and Related Disorders.** Ed. S. E. SALMON. London: W. B. Saunders Company Ltd. 238 pp. £10.75 net.

Multiple myeloma is a relatively common haematological malignancy, but since its discovery in the mid-1800's has engendered substantial clinical and laboratory study. However myeloma at the clinical level remains a complex and unsolved problem. Sydney Salmon as guest editor of this issue of Clinics in Haematology has brought together a talented group of workers to review the clinically relevant areas of research, current treatment, and future prospects.

Disorders related to myeloma, such as amyloidosis, and monoclonal gammopathies of undetermined significance are clearly reviewed by Professor Kyle of the Mayo Clinic. Salmon and Durie review their research work on *in vitro* cloning of myeloma stem cells, kinetic studies, and staging of multiple myeloma, as well as patient management in a series of well written chapters. Barlogie and co-workers discuss their experience using flow cytometry techniques in myeloma research. Other chapters review the immunoregulatory circuits found in myeloma, surface marker studies on circulating lymphocytes, localized plasmacytomas, interferon treatment, and association of acute leukaemia with myeloma.

This latest edition of Clinics in Haematology maintains the high standards we have come to expect from the series. Physicians and research workers interested in multiple myeloma will find this a fascinating and informative review.